# Genetic, virulence, and antimicrobial resistance characteristics associated with distinct morphotypes in ST11 carbapenem-resistant *Klebsiella pneumoniae*

**DOI:** 10.1080/21505594.2024.2349768

**Published:** 2024-05-12

**Authors:** Tao Chen, Yuan Wang, Xiaohui Chi, Luying Xiong, Ping Lu, Xueting Wang, Yunbo Chen, Qixia Luo, Ping Shen, Yonghong Xiao

**Affiliations:** a State Key Laboratory for Diagnosis and Treatment of Infectious Diseases, National Clinical Research Center for Infectious Diseases, Collaborative Innovation Center for Diagnosis and Treatment of Infectious Diseases, the First Affiliated Hospital, Zhejiang University School of Medicine, Hangzhou, Zhejiang, China; b Jinan Microecological Biomedicine Shandong Laboratory, Jinan, Shandong, China

**Keywords:** ST11, morphotype, virulence, chlorhexidine, rpoN

## Abstract

ST11 is the most common lineage among carbapenem-resistant *Klebsiella pneumoniae* (CRKP) infections in Asia. Diverse morphotypes resulting from genetic mutations are associated with significant differences in microbial characteristics among *K. pneumoniae* isolates. Here, we investigated the genetic determinants and critical characteristics associated with distinct morphotypes of ST11 CRKP. An ST11-KL47 CRKP isolate carrying a pLVPK-like virulence plasmid was isolated from a patient with a bloodstream infection; the isolate had the “mcsw” morphotype. Two distinct morphotypes (“ntrd” and “msdw”) were derived from this strain during *in vitro* passage. Whole genome sequencing was used to identify mutations that cause the distinct morphotypes of ST11 CRKP. Transmission electron microscopy, antimicrobial susceptibility tests, growth assays, biofilm formation, virulence assays, membrane permeability assays, and RNA-seq analysis were used to investigate the specific characteristics associated with different morphotypes of ST11 CRKP. Compared with the parental mcsw morphotype, the ntrd morphotype resulted from mutation of genes involved in capsular polysaccharide biosynthesis (*wza*, *wzc*, and *wbaP*), a result validated by gene knockout experiments. This morphotype showed capsule deficiency and lower virulence potential, but higher biofilm production. By contrast, the msdw morphotype displayed competition deficiency and increased susceptibility to chlorhexidine and polymyxin B. Further analyses indicated that these characteristics were caused by interruption of the sigma factor gene *rpoN* by insertion mutations and deletion of the *rpoN* gene, which attenuated membrane integrity presumably by downregulating the phage shock protein operon. These data expand current understanding of genetic, virulence, and antimicrobial resistance characteristics associated with distinct morphotypes in ST11 CRKP.

## Introduction

*Klebsiella pneumoniae* is a crucial nosocomial pathogen that causes severe diseases, including bloodstream infections (BSIs). The considerable ability of carbapenem-resistant *K. pneumoniae* (CRKP) to acquire antibiotic resistance is a major health concern [1]. The rapid spread and increased incidence are being observed globally, presenting an enormous challenge in healthcare settings [2,3]. The dominant clone in China is ST11 CRKP, which produces carbapenem-hydrolyzing *K. pneumoniae* carbapenemase (KPC-2) [4]. Traditionally, CRKP is not considered hypervirulent; nevertheless, in 2016, a fatal outbreak of severe pneumonia caused by ST11 hypervirulent carbapenem-resistant *K. pneumoniae* (hvCRKP) strains occurred in China [5]. The ST11 hvCRKP clone belonged to serotype K47 and harbored a pLVPK-like virulence plasmid carrying the *rmpA2*, *iucABCD*, and *iutA* virulence genes. Since then, emergence of ST11 hvCRKP infections has become a recent public health crisis [6–8].

Clinical *K. pneumoniae* strains usually form glistening mucoid colonies on the culture plate. In general, the mucoid morphology, in comparison with nonmucoid isolates, is more resistant to phagocytosis, and is associated with virulence in animal experiments [9]. Hypervirulent *K. pneumoniae* (HvKp) colonies typically present with a hypermucoviscous morphology, which is presumably associated with over-expression of capsular polysaccharides [10]. A previous study reported that during frequent subculture on medium, distinct colonial morphologies were obtained from the parent mucoid colony. Nevertheless, the clinical implications of this microbial characteristic were not clearly defined [11]. In addition, He et al. investigated the *in vivo* evolution of the mucoid phenotype of ST11-KL64 CRKP, and found opposite evolution of pathogenicity driven by the *wzc* and *wcaJ* mutations [12]. Liu et al. reported that ST15 CRKP showing the clinical “rdar” (red, dry, and rough) morphology resulting from the *bcsA* mutation was able to better adapt to the hospital environment than ST15 CRKP with the more common “mcsw” (mucoid, clean margin, smooth and wet surface) morphology [13]. These data indicate that the diverse morphotypes resulting from mutations are associated with significant differences in certain microbial characteristics.

Here, we obtained an ST11-KL47 BSI-CRKP clinical isolate carrying a pLVPK-like virulence plasmid and investigated its diverse morphotypes. Whole genome sequencing (WGS), gene knockout, antimicrobial susceptibility tests, pairwise competition, biofilm formation, virulence, membrane permeability assays, RNA-seq, and RT-qPCR were performed to identify the genetic determinants and critical characteristics associated with different morphotypes.

## Materials and methods

### Bacterial isolates and antimicrobial susceptibility testing (AST)

A carbapenem-resistant *K. pneumoniae* (CRKP) clinical isolate, KP10042, with the “mcsw” morphotype was isolated from a blood culture obtained from an in-patient at a 2,500-bed tertiary care hospital in China. Two distinct morphological phenotypes “ntrd” (non-mucoid morphology with translucent, rough colonies, and a dry surface) and “msdw” (mucoid morphology with smaller, dewdrop colonies, and a wet surface) were derived from KP10042. The agar dilution method was used to determine the minimum inhibitory concentrations (MICs) of most antibiotics and disinfectants, and the broth microdilution method was used to test for resistance to polymyxin B (PMB) and tigecycline in accordance with CLSI guidelines [14]. Interpretation of results was based on CLSI breakpoints (the exception was tigecycline, which was assessed according to the criteria of the USA FDA). All protocols were approved by the institutional review board at the First Affiliated Hospital, Zhejiang University School of Medicine, China (approval no. 2017–442).

### Whole genome sequencing and bioinformatics analysis

Four colonies of each morphotype were selected at random and sequenced using an Illumina Hiseq2500 instrument. *De novo* assembly of the short-read data was performed by SPAdes [15]. MinION sequencing was performed on the initial isolate KP10042. Complete genomes were generated by Unicycler [16], and annotated on the RAST server. K-type and O-type were identified by Kleborate [17]. Antimicrobial resistance genes were identified using the CARD [18] and Resfinder (http://genepi.food.dtu.dk/resfinder), while virulence factors were identified through the VFDB [19] (accessed on 19 November 2023). Plasmid replicons were identified using PlasmidFinder [20]. All *de novo* assemblies were aligned to the reference genome of *K. pneumoniae* KP10042 using MUMmer [21] to acquire pairwise nucleotide differences. To predict whether the identified amino acid substitutions affect protein function, the PROVEAN software was used (https://www.jcvi.org/research/provean). PCR and Sanger sequencing were performed to further determine the IS type inserted in the *rpoN* and the *cps* genes (capsular polysaccharide biosynthesis) using the primers shown in Table S1.

### Gene knockout and mutant construction

Mutations were constructed using the λ Red recombinase system, as previously described [22]. The mutagenesis plasmid pACBSR carries the genes encoding Red recombinase under the control of the arabinose promoter. Regions (approximately 0.5 kb) flanking the upstream and downstream sequences of the target gene were amplified using specific primers, with KP10042 as the template (Table S1). The apramycin resistance gene was amplified from pCAP03-acc(3)IV, which contains FRT sites to permit subsequent excision of the apramycin cassette. Excision of the apramycin cassette from the chromosome was performed using the helper plasmid pFLP.

### Biofilm formation assay

The biofilm formation assay was performed as described previously [23], with slight modifications. Briefly, overnight cultures were diluted 1:100 in fresh LB broth, and 200 μL of aliquots were transferred to 96-well polystyrene plates in six replicates. The plates were incubated at 37°C for 24 h and the wells were washed three times with PBS, followed by staining with 0.1% crystal violet at room temperature. Next, 200 μL of 95% ethanol was added to solubilize the bound dye in the wells, and the absorbance at OD_595_ was measured.

### Growth curve assay

Growth was measured in an antibiotic-free environment, as described previously [24] but with some modifications. Briefly, bacterial suspensions (10^8^ CFU/mL) were diluted 1:100 in fresh LB broth. A 200 μL aliquot of each sample was then transferred to a 96-well microtiter plate and incubated at 37°C for 1 day. The absorbance of each sample at OD_600_ was measured every 10 min.

### Serum resistance assay

Serum resistance was determined by measuring growth curves in LB broth containing 10% pooled normal human serum, as previously described [25]. The strains were grown overnight until saturation, and then diluted 1:100 in LB broth containing 10% human serum. Growth curves were obtained by measuring the OD_600_ every 10 min for 24 h.

### *In vitro* competition assay

Pairwise competition experiments were used to estimate the relative fitness of isolates with different morphotypes. Cultures of each different morphotype were diluted to 0.5 McFarland turbidity, mixed at a ratio of 1:1 (at 0 h) and then added into 4 mL of LB broth. The mixture was incubated at 37°C for 24 h with shaking. Dilutions of the mixture were plated on LB agar at 0 and 24 h, and bacteria were identified morphologically and counted. Selection Competition Index (CI) values were calculated as the mean ratio of the CFU.

## Transmission electron microscopy (TEM)

TEM was performed at the Center of Cryo-Electron Microscopy, Zhejiang University. Briefly, a pellet of each sample was fixed with 2.5% glutaraldehyde/PBS at 4°C, washed in PBS, post-fixed for 1 h with 1% OsO_4_ in PBS, and then washed again with PBS. After fixation, the sample was dehydrated by passage through graded ethanol solutions. The specimen was then incubated with a mixture comprising different ratios of absolute acetone and the final Spurr resin. Then the samples were transferred to Spurr resin mixture overnight, embedded in Spurr resin, and polymerized at 70°C for 12 h. Finally, the specimens were sectioned using a LEICA EM UC7 ultratome and examined under a Tecnai G2 spirit 120kV TEM.

### Mucoviscosity assay

The bacterial cultures were grown overnight in LB broth and centrifuged for 5 min at 2000 rpm. Supernatant (200 μL) was placed in 96-well polystyrene plates and the OD_600_ was measured.

### Virulence assay

All animal experiments were approved by the Institutional Animal Care and Ethics Committee at the First Affiliated Hospital of Zhejiang University, School of Medicine (reference number: 2022–835). *Galleria mellonella* and mouse intraperitoneal infection models were utilized, as previously described [26,27] but with slight modifications. Briefly, ten larvae, each weighing approximately 250 mg, were selected randomly and tested for each isolate. Subsequently, 20 μL of bacterial suspension (10^6^ CFU/mL in PBS) was injected into the second left proleg. The insects were then incubated at 37°C and observed at 24 h intervals. For the mouse model, 5-week-old BALB/c female mice were injected intraperitoneally with 1 × 10^7^ CFU of each isolate, resuspended in 100 μL of PBS (each group contained 10 mice). After inoculation, the physical condition of each mouse was monitored and recorded every 12 h.

### Membrane permeability assay

The membrane permeability assay was performed as described previously [28], with slight modifications. Cultures of wild-type and Δ*rpoN* strains were diluted to 0.5 McFarland turbidity and then diluted 1:100 in fresh LB broth containing 1% sodium dodecyl sulfate (SDS) and 100 μg/mL polymyxin B nonapeptide (PMBN) (both of which compromise the integrity of bacterial cell membrane). Absorbance at OD_600_ was measured every 10 min for 5 h.

### RNA-seq analysis and RT-qPCR

Total RNA was extracted from overnight cultures of KP10042-Δ*rpoN* and KP10042-WT. RNA-seq was performed on an Illumina HiSeqTM 4000. After trimming the raw reads, the clean data were mapped to the reference genome of *K. pneumoniae* KP10042 using Hisat2. Differentially expressed genes (DEGs) were identified using DESeq2, with the following thresholds: absolute log_2_fold change > 1; false discovery rate (FDR) <0.01. Gene enrichment analysis was performed by clusterProfiler [29] using KEGG and GO Term gene sets. Relative expression of the *pspA*, *pspB*, *pspC*, *pspD*, and *pspG* genes was verified by RT-qPCR, and normalized to that of the *gapA* reference gene using the 2 ^−ΔΔCt^ method. The primers used for RT-qPCR are listed in Table S1.

### Statistical analysis

All experiments were performed with at least three replicates. Continuous data are expressed as the mean ± standard deviation (mean ± SD), and comparisons were made using Student’s t-test. A P-value <0.05 (two-tailed) was considered significant. Mortality of *G. mellonella* and mice was assessed by Kaplan-Meier analysis and the log-rank test.

## Results

### Characterization of the carbapenem-resistant *K. pneumoniae* strain KP10042

On 12 April 2013, a patient (aged 67 years) was admitted to the infectious diseases department of the First Affiliated Hospital, Zhejiang University School of Medicine, due to pneumonia. A CRKP isolate (KP10042) was detected repeatedly in sputum samples, and was isolated from a blood culture, during hospitalization. Due to extensive drug resistance, the patient received tigecycline and piperacillin-tazobactam, but infection control was poor and *K. pneumoniae* was detected repeatedly in blood cultures. Unfortunately, the patient died from a severe lung infection and septic shock.

Genome sequencing revealed that the CRKP KP10042 strain belonged to ST11-KL47. KP10042 harbored five beta-lactamase genes (including *bla*_KPC-2_, *bla*_TEM-1B_, *bla*_CTX-M-14b_, *bla*_CTX-M-65_, and *bla*_SHV-182_), four genes associated with aminoglycoside resistance (*aac(6’)-Ib-cr, rmtB, aadA16*, and *aadA2b*), and other resistance genes (*dfrA27*, *sul1*, *catA2*, *floR*, *fosA3*, and *ARR-3*). The isolate also carried virulence factors associated with hypervirulence, which are typically mobilized by virulence plasmids or integrative and conjugative elements such as the genes associated with hypermucoid phenotype *rmpA2*, aerobactin (*iucABCDiutA*), and yersiniabactin (*fyuA, irp1, irp2*, and *ybtAEPQSTUX*). KP10042 was resistant to the majority of antimicrobial agents tested, but was susceptible to PMB and tigecycline (Table 1).

**Table 1.** Susceptibility of isolate KP10042, its variants, and the corresponding knockout strains, to commonly used antibiotics and disinfectants.

Isolate	Antibiotic (μg/ml)													Disinfectant (μg/ml or %)			
	CAZ	FEP	TZP	IPM	MEM	LVX	CIP	AMP	AMK	GEN	CSL	SXT	PMB	TGC	CHX	SHC (%)	BK
KP10042	>64	32	>128/4	8	4	>16	>32	>128	>64	>128	>128/64	>8/152	0.5	0.5	64	0.25	32
KP10042 ntrd-1	>64	32	>128/4	8	4	>16	>32	>128	>64	>128	>128/64	>8/152	0.5	0.5	64	0.25	32
KP10042 ntrd-2	>64	32	>128/4	8	4	>16	>32	>128	>64	>128	>128/64	>8/152	0.5	0.5	64	0.25	32
KP10042 ntrd-3	>64	32	>128/4	8	4	>16	>32	>128	>64	>128	>128/64	>8/152	0.5	0.5	64	0.25	32
KP10042 ntrd-4	>64	32	>128/4	8	4	>16	>32	>128	>64	>128	>128/64	>8/152	0.5	0.5	64	0.25	32
KP10042 msdw-1	>64	32	>128/4	8	4	>16	>32	>128	>64	>128	>128/64	>8/152	0.25	0.5	16	0.25	16
KP10042 msdw-2	>64	32	>128/4	8	4	>16	>32	>128	>64	>128	>128/64	> 8/152	0.25	0.5	16	0.25	32
KP10042 msdw-3	>64	32	>128/4	8	4	>16	>32	>128	>64	>128	>128/64	>8/152	0.25	0.5	16	0.25	16
KP10042 msdw-4	>64	32	>128/4	8	4	>16	>32	>128	>64	>128	>128/64	>8/152	0.25	0.5	16	0.25	32
KP10042 ntrds-1	>64	32	>128/4	8	4	>16	>32	>128	>64	>128	>128/64	>8/152	0.25	0.5	16	0.25	32
KP10042 ntrds-2	>64	32	>128/4	8	4	>16	>32	>128	>64	>128	>128/64	>8/152	0.25	0.5	16	0.25	16
KP10042 ntrds-3	>64	32	>128/4	8	4	>16	>32	>128	>64	>128	>128/64	>8/152	0.25	0.5	16	0.25	32
KP10042 ntrds-4	>64	32	>128/4	8	4	>16	>32	>128	>64	>128	>128/64	>8/152	0.25	0.5	16	0.25	32
KP10042 ∆*wza*	>64	32	>128/4	8	4	>16	>32	>128	>64	>128	>128/64	>8/152	0.5	0.5	64	0.25	32
KP10042 ∆*wzc*	>64	32	>128/4	8	4	>16	>32	>128	>64	>128	>128/64	>8/152	0.5	0.5	64	0.25	32
KP10042 ∆*wbaP*	>64	32	>128/4	8	4	>16	>32	>128	>64	>128	>128/64	>8/152	0.5	0.5	64	0.25	32
KP10042 ∆*rpoN*	>64	32	>128/4	8	4	>16	>32	>128	>64	>128	>128/64	>8/152	0.25	0.5	16	0.25	32

Abbreviations: CAZ, ceftazidime; FEP, cefepime; TZP, piperacillin-tazobactam; IPM, imipenem; MEM, meropenem; LVX, levofloxacin; CIP, ciprofloxacin; AMP, ampicillin; AMK, amikacin; GEN, gentamicin; CSL, cefoperazone-sulbactam; SXT, sulfamethoxazole-trimethoprim; PMB, polymyxin B; TGC, tigecycline; CHX, Chlorhexidine; SHC, sodium hypochlorite; BK, benzalkonium bromide.

Genomic analysis revealed that KP10042 carries four plasmids in total (Figure S1). Plasmid 1 (pKP10042–1) is a 178 kb IncFIB-IncHI1B type virulence plasmid carrying genes associated with hypermucoid phenotype *rmpA2* and iron acquisition systems (*iucABCDiutA*). The plasmid is 99.9% identical (100% coverage) to the previously reported pVir-CR-HvKP4 (accession number MF437313.1) detected in the hvCRKP strain CR-HvKP4, which was recovered during a fatal outbreak in a Chinese hospital [5]. Plasmid 2 (pKP10042–2) is a 169 kb IncFII-R multidrug resistance plasmid that carries a variety of resistance determinants, including *rmtB*, *bla*_CTX-M-65_, *bla*_TEM-1B_, *fosA3*, and *catA2*, as well as the carbapenemase gene *bla*_KPC-2_. It is 99.9% identical to pKPC-CR-HvKP4 (accession number MF437312.1), with 97% coverage. Plasmid 3 (pKP10042–3) is a 117 kb IncI1-I plasmid harboring multiple resistance genes, including *bla*_CTX-M-14b_, *aadA16, aac(6’)-Ib-cr*, *sul1*, *dfrA27*, *ARR-3*, and *floR*. In addition to the above three plasmids, KP10042 harbors a 12 kb ColRNAI plasmid (pKP10042–4) that does not carry resistance genes or virulence determinants. These findings suggest that KP10042 might be a hvCRKP isolate.

### *In vitro* passage results in KP10042 with two distinct morphologies

**Figure 1.**
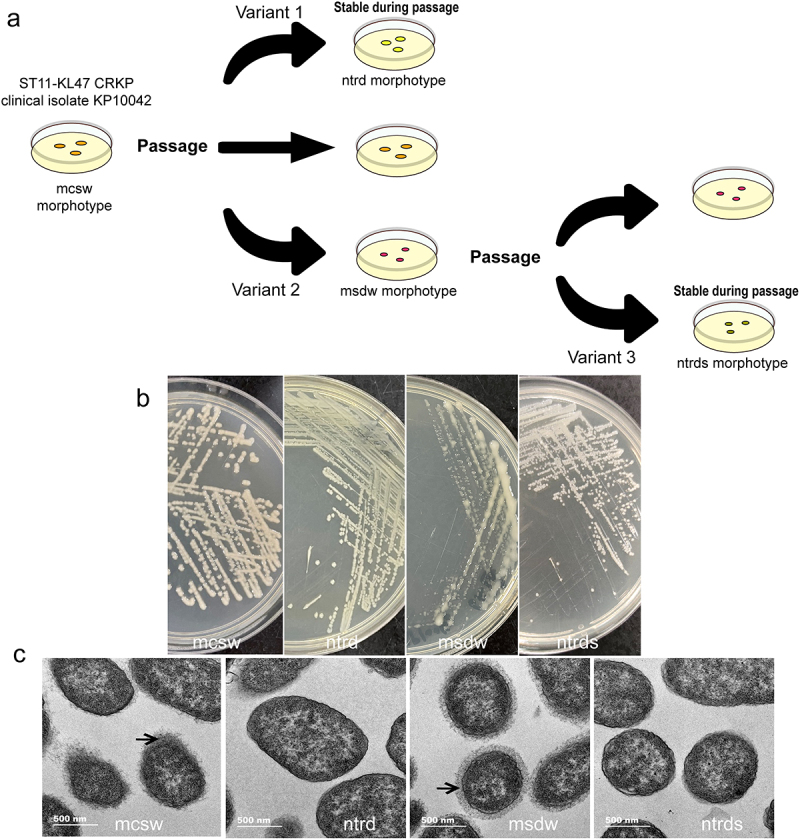
Four variants with distinct morphologies derived from ST11-KL47 CRKP isolate KP10042. (a) A schematic showing the appearance of variants with distinct morphologies during *in vitro* passage. (b) Colony morphology of variants on LB agar plates. (c) Transmission electron microscopy images of the variants with different morphotypes. Arrows point to the capsule.

On LB plates, the clinical isolate KP10042 exhibited a mucoid morphology, with a raised center, a clean margin, and a smooth and wet surface, named the “mcsw” morphotype [13]. Frequent subculture of the KP10042 isolate in LB broth resulted in two variants, each with a distinct colonial morphology. Variant 1 exhibited a non-mucoid morphology with translucent, rough colonies, and a dry surface on LB agar; this was named the “ntrd” morphotype. Variant 2 exhibited a mucoid morphology with smaller, dewdrop colonies, and a wet surface, and was named the “msdw” morphotype (Figure 1(a,b)).

To determine whether these morphotypes were stable, both were serially passaged, daily for 10 days, in antibiotic-free LB broth. After subculture on an antibiotic-free LB plates, the ntrd morphotype was stable; however, the msdw variant was volatile and generated a novel subpopulation, which displayed a “ntrd-like” morphotype but with smaller colonies; we named this the “ntrds” morphotype (Figure 1(a,b)).

### The ntrd and msdw morphologies are associated with different characteristics

**Figure 2.**
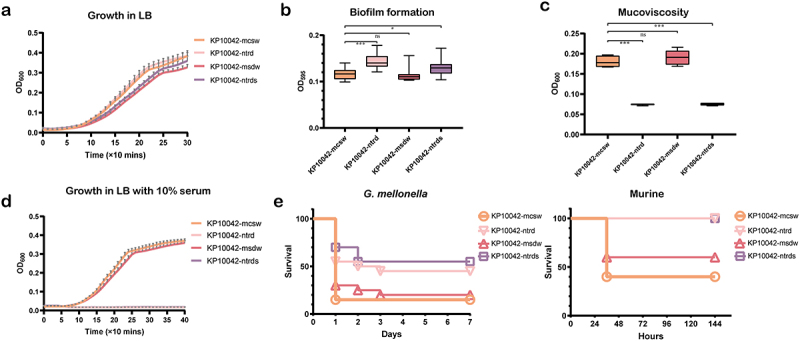
Phenotypic differences between variants with distinct morphologies. (a) Growth curves of the variants in LB broth. (b) Biofilm formation. (c) Mucoviscosity of the different morphotypes. (d) Serum resistance of the different morphotypes. Differences in serum resistance were determined by comparing growth curves in LB broth with those in LB broth supplemented with 10% pooled normal human serum. (e) Survival curves generated by the *G. mellonella* infection model and the murine intraperitoneal infection model. Ten larvae per group were injected with 20 μL (10^6^ CFU/mL) of each morphotype. Ten mice of each group were injected intraperitoneally with 10^7^ CFU of each morphotype. Survival was monitored for 7 days.

To investigate the phenotypic characteristics of the different morphotypes, we selected four colonies of each at random and conducted a series of assays. Micromorphology was examined by TEM, which showed that the mcsw and msdw morphotypes have a very thick capsule, whereas the ntrd and ntrds morphotypes are nonencapsulated (Figure 1(c)). We then asked whether the different morphotypes affect bacterial growth. The msdw and ntrds morphotypes grew more slowly than the mcsw and ntrd morphotypes (Figure 2(a)). Next, we performed *in vitro* competition growth experiments to compare the relative fitness of the morphotypes. The results of a competition assay showed that the ntrd morphotype had a competitive advantage over the mcsw morphotype, while the msdw morphotype was out-competed by the mcsw morphotype (Table S2). We then evaluated whether different morphologies affect biofilm formation. Compared with the mcsw morphotype, the ntrd and ntrds morphotypes exhibited stronger biofilm formation, whereas that by msdw was similar to mcsw (Figure 2(b)). The TEM results indicated that the ntrd-like morphotype might be associated with capsule deficiency; therefore, we evaluated the mucoviscosity, serum resistance, and virulence of the morphotypes. Based on the absorbance (OD_600_) of the supernatants, the mucoviscosity of the ntrd and ntrds morphotypes was much lower than that of the mcsw and msdw morphotypes, and they failed to grow when cultured for 6 h in LB broth supplemented with 10% human serum (Figure 2(c–d)). In both *in vivo* models, mortality caused by the mcsw and msdw morphotypes was comparable (approximately 50% of infected mice, and 20% of infected larvae, survived); both of these morphotypes were more lethal than the ntrd and ntrds morphotypes (all mice, and approximately 50% of larvae, survived) (Figure 2(e)).

In addition, to explore whether morphotype is associated with resistance to antibiotics and disinfectants, we measured the MICs of commonly used antibiotics and disinfectants. Compared with parental KP10042, the ntrd morphotype showed the same MICs for all tested antibiotics and disinfectants; however, the msdw and ntrds morphotypes exhibited a 4-fold and 2-fold reduction in the MICs of chlorhexidine and PMB, respectively (Table 1).

### The ntrd and msdw morphotypes result from mutations in the *cps* genes and the sigma factor gene *rpoN*, respectively

To identify the genetic determinants underlying the diverse morphotypes, we first performed genomic sequencing and comparative genome analysis on four colonies of each morphotype. The results (Table 2) revealed that the mutated genes *wza*, *wzc*, and *wbaP*, which encode proteins involved in capsular polysaccharide biosynthesis, were associated with the ntrd and ntrds morphotypes. An insertion sequence, ISKpn74/ISKpn26, was detected in the *wza* or *wzc* genes in all four KP10042-ntrd isolates, whereas *wbaP* and *wzc* were disrupted by ISs [*wzc*::ISKpn26, *wbaP*::ISKpn26] in two of the ntrds isolates, and the SNPs *wbaP* T854A (amino acid I285K) and G292T (leading to a premature termination codon TAA) were detected in the remaining two ntrds isolates. According to the PROVEAN scores, the mutation I285K in WbaP was predicted to be deleterious. Any one of these mutated genes might cause capsule deficiency in *K. pneumoniae*. By contrast, the gene *rpoN* (RNA polymerase factor sigma-54, σ54) in all of the msdw and ntrds isolates was disrupted by a transposon [*rpoN*::ISKpn18], which might account for the switch to the msdw morphotype. Thus, the ntrds morphotype might be generated by mutations in the *cps* and *rpoN* genes.

**Table 2.** Description of the isolates used in this study.

Strain	Description	IS direct repeat	Morphotype
KP10042	Clinical isolate with wild-type KL47 and *rpoN*	–	mcsw
KP10042 ntrd-1	Isogenic mutant of KP10042 with mutated *wza* (KP10042 *wza*::ISKpn74 at 653) and wild-type *rpoN*	tggtaaaga	ntrd
KP10042 ntrd-2	Isogenic mutant of KP10042 with mutated *wzc* (KP10042 *wzc*::ISKpn26 at 312) and wild-type *rpoN*	ttag	ntrd
KP10042 ntrd-3	Isogenic mutant of KP10042 with mutated *wzc* (KP10042 *wzc*::ISKpn26 at 753) and wild-type *rpoN*	cttg	ntrd
KP10042 ntrd-4	Isogenic mutant of KP10042 with mutated *wza* (KP10042 *wza*::ISKpn26 at 573) and wild-type *rpoN*	ttaa	ntrd
KP10042 msdw-1	Isogenic mutant of KP10042 with wild-type KL47 and mutated *rpoN* (KP10042 *rpoN*::ISKpn18 at 670)	caa	msdw
KP10042 msdw-2	Isogenic mutant of KP10042 with wild-type KL47 and mutated *rpoN* (KP10042 *rpoN*::ISKpn18 at 670)	caa	msdw
KP10042 msdw-3	Isogenic mutant of KP10042 with wild-type KL47 and mutated *rpoN* (KP10042 *rpoN*::ISKpn18 at 192)	gag	msdw
KP10042 msdw-4	Isogenic mutant of KP10042 with wild-type KL47 and mutated *rpoN* (KP10042 *rpoN*::ISKpn18 at 995)	tct	msdw
KP10042 ntrds-1	Isogenic mutant of KP10042 with mutated *wzc* (KP10042 *wzc*::ISKpn26 at 1440) and mutated *rpoN* (KP10042 *rpoN*::ISKpn18 at 670)	ctag caa	ntrds
KP10042 ntrds-2	Isogenic mutant of KP10042 with mutated *wbaP* (KP10042 *wbaP* I285K) and mutated *rpoN* (KP10042 *rpoN*::ISKpn18 at 670)	caa	ntrds
KP10042 ntrds-3	Isogenic mutant of KP10042 with mutated *wbaP* (KP10042 *wbaP*::ISKpn26 at 807) and mutated *rpoN* (KP10042 *rpoN*::ISKpn18 at 670)	ctag caa	ntrds
KP10042 ntrds-4	Isogenic mutant of KP10042 with mutated *wbaP* (KP10042 *wbaP* E98X) and mutated *rpoN* (KP10042 *rpoN*::ISKpn18 at 670)	caa	ntrds
KP10042 ∆*wza*	In-frame deletion of *wza* in KP10042	–	ntrd
KP10042 ∆*wzc*	In-frame deletion of *wzc* in KP10042	–	ntrd
KP10042 ∆*wbaP*	In-frame deletion of *wbaP* in KP10042	–	ntrd
KP10042 ∆*rpoN*	In-frame deletion of *rpoN* in KP10042	–	msdw

To validate whether these two morphotypes (ntrd and msdw) result from mutations in the *wza*, *wzc*, *wbaP*, and/or *rpoN* genes, we performed a knockout experiment. To do this, we engineered isogenic mutants KP10042Δ*wza*, KP10042∆*wzc*, KP10042∆*wbaP*, and KP10042Δ*rpoN*. In-frame deletion of any of the *wza*, *wzc*, and *wbaP* genes in KP10042 converted the mcsw morphotype to the ntrd morphotype (Fig. S2a). TEM analyses demonstrated that all of the KP10042Δ*wza*, KP10042∆*wzc*, and KP10042∆*wbaP* mutants were nonencapsulated (Fig. S2b), indicating that capsule deficiency was responsible for the switch to the ntrd morphotype, and was attributable to mutation of genes *wza*, *wzc*, or *wbaP*. As expected, deletion of *wza*, *wzc*, or *wbaP* from KP10042 reduced the virulence potential, but increased biofilm production, compared with the wild-type strain (Fig. S2c – g). By contrast, KP10042Δ*rpoN* displayed a msdw morphotype and slower growth, but did not cause capsule loss or attenuate virulence (Fig. S2). Furthermore, and as expected, KP10042Δ*rpoN* displayed increased susceptibility to chlorhexidine and PMB (Table 1). These data indicate that mutated *rpoN* causes the msdw morphotype and contributes to increased susceptibility to chlorhexidine and PMB.

### Increased susceptibility of the *rpoN* mutant to chlorhexidine and PMB is associated with attenuated membrane integrity

**Figure 3.**
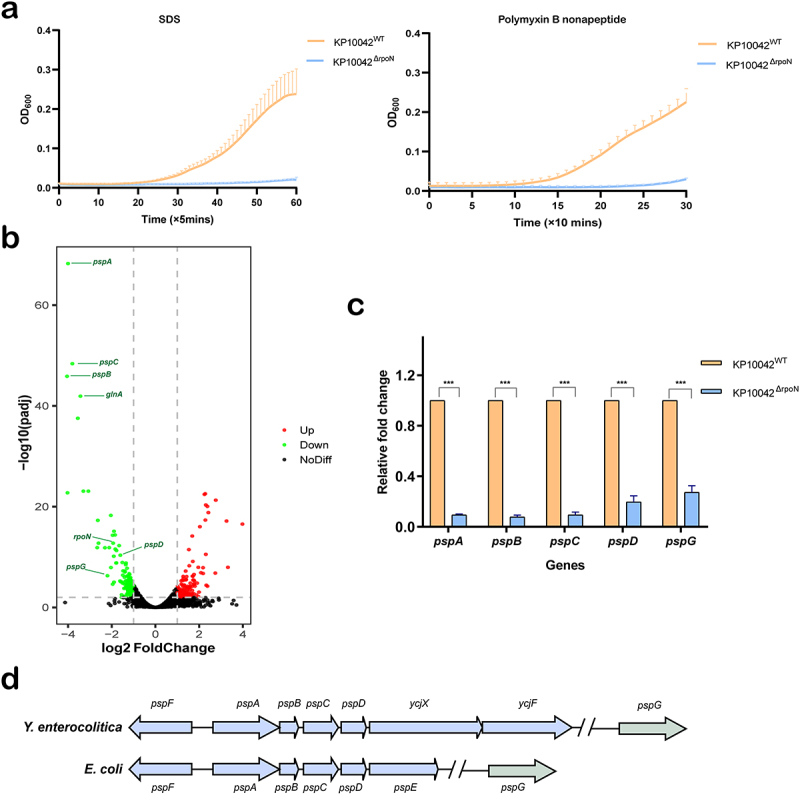
Increased susceptibility of the msdw morphotype to chlorhexidine and PMB is associated with attenuated membrane integrity. (a) Comparison of membrane permeability of the wild-type and Δ*rpoN* strains in an assay using 1% sodium dodecyl sulfate (SDS) and 100 μg/mL polymyxin B nonapeptide (PMBN) (both of which compromise the integrity of bacterial membrane). (b) Volcano plot of differentially expressed genes. The red and green dots denote significantly upregulated and downregulated genes, respectively, and the black dots represent non-differentially expressed genes. Representative genes are depicted. (c) Downregulated transcription of the psp operon in the Δ*rpoN* mutant, validated by RT-qPCR. (d) A diagram of the psp operon in different species.

It is worth noting that chlorhexidine and PMB work by disrupting membrane integrity, which generally increases membrane permeability and causes bacterial cell death [30,31]. Therefore, we hypothesized that increased susceptibility of the msdw morphotype to these agents was associated with reduced membrane integrity. To verify this, we utilized SDS and PMBN (both of which compromise the integrity of the bacterial membrane) in membrane permeability assays. Compared with the wild-type strain, the Δ*rpoN* mutant did not grow in the presence of 1% SDS or 100 μg/mL PMBN over a 6 h period (Figure 3(a)), indicating that deletion of the *rpoN* gene is associated with attenuated membrane integrity.

To better understand the mechanism underlying increased susceptibility of the Δ*rpoN* mutant to chlorhexidine and PMB, we performed transcriptomic analysis of wild-type and Δ*rpoN* cells using RNA-seq. We identified a total of 376 DEGs, 150 upregulated and 226 downregulated, in the Δ*rpoN* mutant (Figure 3(b)). By combining GO enrichment analysis and DEG -fold changes, we identified five potent hits: *pspA*, *pspB, pspC, pspD*, and *pspG*. The transcriptomic results of these genes were validated by qRT-PCR, the results of which demonstrated that the transcriptional levels of these genes were downregulated significantly in the Δ*rpoN* mutant (Figure 3(c)). The phage shock protein (psp) operon of *K. pneumoniae* is arranged as *pspF-pspABCDycjXF*, which is identical to that of *Y. enterocolitica*, but different from *pspABCDE* in *E. coli*. All species carry the unlinked gene *pspG* (Figure 3(d)). The psp response mediated by the psp operon is important for maintaining the membrane permeability barrier [32], and expression of the psp operon induced by assault on the cell membrane is dependent on RpoN (σ54) [33,34]. Therefore, in accordance with these observations, we concluded that the lack of RpoN in *K. pneumoniae* presumably mediates marked downregulation of the psp operon, further leading to a reduced ability to maintain membrane integrity, as well as increased susceptibility to membrane disruptors (i.e. chlorhexidine and PMB).

### The prevalence of distinct morphotypes among ST11 CRKP strains

We gathered clinical ST11 BSI-CRKP strains from this hospital between 2013 and 2017, considering only the initial blood isolate per patient. To ascertain the proportion of each morphotype, we retrospectively screened 154 ST11 BSI-CRKP isolates, revealing that only two morphotypes were detected in the ST11 BSI-CRKP population: mcsw (139/154), and ntrd (15/154). None of the strains displayed the msdw morphotype.

## Discussion

Single-celled organisms have small genomes; therefore, their genomic storage capability is limited. To compensate, these organisms have evolved strategies to modify their genome and generate physiological diversity to respond to their immediate surroundings [35]. This can be achieved through recombination, duplication, point mutations, and mobile genetic elements (especially ISs). For example, ST258 CRKP has two opposing infection programs acquired through gain- and loss-of-function mutations in the genes responsible for capsule biosynthesis [36].

In 2013, we isolated a BSI-causing ST11-KL47 CRKP strain, KP10042, harboring a pLVPK-like virulence plasmid from a patient; this was before the first report of an outbreak of ST11-KL47 hvCRKP in a Chinese hospital in 2016 [5]. The genome of KP10042, which has a mcsw morphology, is highly homologous with that of hvCRKP strain CR-HvKP4 [5], and ultimately caused the death of the patient. The mcsw morphotype seems to be predominant among CRKP strains in China [14], and the results of the present study confirm this. Interestingly, two variants with distinct morphologies were obtained after frequent subculture in LB broth; these were named the ntrd and msdw morphotypes. The phenotype of ntrd was similar to that previously reported for the “matt” phenotype of ST14-KL64 *K. pneumoniae*, which is significantly less virulent than the mucoid phenotype [37]. Likewise, the ntrd morphotype displayed attenuated virulence, but increased biofilm production, compared with the mcsw morphotype. Further analyses, including gene knockout and phenotypic tests, indicated that the ntrd morphotype was caused by capsule deficiency mediated by point mutations, or disruption of the *cps* genes by diverse ISs (include ISKpn74 and ISKpn26). A recent study by Ye et al. also indicated that deletion of the acyltransferase gene (*act*) from the *cps* locus plays a crucial role in the evolution of virulence of ST11 CRKP [38]. The capsule is a critical virulence factor, and was shown to be costly in nutrient-rich media in a study by Buffet et al. [39]. Capsule inactivation may be a way of alleviating metabolic costs, and loss of the capsule may improve accessibility to surface adhesin proteins [40]; therefore, the ntrd morphotype was more adaptive in LB broth, and displayed increased biofilm production.

By contrast, the msdw morphotype grew more slowly, was less competitive, and showed increased susceptibility to chlorhexidine and PMB, suggesting that it might be defective; it is of note that none of the clinical BSI-CRKP isolates had the msdw morphotype. Genomic comparison analysis and gene knockout studies indicated that the msdw morphotype was caused by interruption of the *rpoN* gene, encoding RNA polymerase sigma factor σ54, by ISKpn18. In *Klebsiella* spp. and *E. coli*, σ54 (RpoN) works in conjunction with the NtrC class transcriptional activator GlnG to regulate expression of *glnA* (encoding glutamine synthetase) and the Ntr (nitrogen regulation) regulon in response to nitrogen limitation [41,42]. In pathogenic bacteria, *rpoN* controls expression of virulence factors such as flagellin (in *Vibrio anguillaru*) and pilin (in *Pseudomonas aeruginosa*) [43,44]. These data demonstrate that the function of RpoN in distinct species is likely diverse.

Inactivation of the *rpoN* gene clearly increases the resistance of *S. enterica* to PMB; however, the underlying mechanism is unknown [45]. By contrast, we found that interruption of *rpoN* mediated a 2-fold reduction in the MIC of PMB for ST11 *K. pneumoniae*. In accordance with the results of the RNA-seq assay, increased susceptibility to PMB was independent on PmrA/PhoP (data not shown). Moreover, some authors report *K. pneumoniae* showing cross-resistance to polymyxin and chlorhexidine [46,47]. Likewise, our data also indicate that the Δ*rpoN* mutant shows increased susceptibility to chlorhexidine and PMB, both of which are membrane-permeabilizing agents. Furthermore, membrane permeability assays revealed that deleting *rpoN* attenuated membrane integrity. In accordance with the RNA-seq results and a literature search, we have made an assumption regarding the mechanism underlying increased susceptibility of the Δ*rpoN* mutant to chlorhexidine and PMB; however, this assumption was not confirmed in the laboratory. The precise mechanism remains unclear and warrants further investigation.

## Conclusion

This represents the first comprehensive characterization of genetic, virulence, and antimicrobial resistance characteristics associated with distinct morphotypes in ST11 CRKP. Our study underscores the significance of the capsule, particularly in the context of ST11 CRKP infections that are challenging to eradicate. Additionally, we have identified that the disruption of the sigma factor gene rpoN leads to a novel morphotype and enhances the susceptibility of ST11 CRKP to chlorhexidine and PMB. These findings significantly augment our current understanding of the genetic determinants and resistance characteristics linked to distinct morphotypes.

## Supplementary Material

Supplemental Material

## Data Availability

The genome sequences of all *K. pneumoniae* strains were uploaded to GenBank with accession number PRJNA947949. The raw data of RNA-seq was uploaded to SRA with accession numbers PRJNA1016256.
