# Supplementary material for: Genetic, virulence, and antimicrobial resistance characteristics associated with distinct morphotypes in ST11 carbapenem-resistant Klebsiella pneumoniae
Source: Virulence. 2024 May 12;15(1):2349768. doi: 10.1080/21505594.2024.2349768 (PMC11093053; doi:10.1080/21505594.2024.2349768)
Supplement: Supplemental Material [file KVIR_A_2349768_SM6163.zip › Table S1 plasmids primers .docx]

**Table S1. Plasmids and primers used in this study**

| **Plasmid/ Primers** | **Description/ Sequence** | **Use** |
| --- | --- | --- |
| pACBSR-Hyg | A p15A replicon plasmid containing an arabinose-inducible λ-Red recombinase and hygromycin resistance selection marker | Homologous recombination |
| pCAP03-acc(3)IV | Template for amplification of the apramycin resistance cassette | Amplification of apramycin cassette |
| pFLP-Hyg | Plasmid containing a heat-shock inducible FLP recombinase and hygromycin resistance selection marker | Excision of apramycin cassette |
|  |  |  |
| wzaL-F | tatcgggttgtaccattatccctgg | Amplification of wza upstream for wza deletion |
| wzaL-R | atattggtaataggttgctggcc | Amplification of wza upstream for wza deletion |
| aprR-F(wza) | cagcaacctattaccaatatattccggggatccgtcgacc | Amplification of apramycin cassette |
| aprR-R(wza) | ccttggtacaaagagaatatctcctgtaggctggagctgcttcg | Amplification of apramycin cassette |
| wzaR-F | cgaagcagctccagcctacaggagatattctctttgtaccaagg | Amplification of wza downstream for wza deletion |
| wzaR-R | ttcgtaccagccgaatcaatcac | Amplification of wza downstream for wza deletion |
| rpoNL-F | aattgctccagcttgctgaacttcg | Amplification of rpoN upstream for rpoN deletion |
| rpoNL-R | accctgctgcgcgtcagc | Amplification of rpoN upstream for rpoN deletion |
| aprR-F(rpoN) | cggctgacgcgcagcagggtattccggggatccgtcgacc | Amplification of apramycin cassette |
| aprR-R(rpoN) | acgaggtagatgtaggctggagctgcttcg | Amplification of apramycin cassette |
| rpoNR-F | ccagcctacatctacctcgtcgtgaagatcgg | Amplification of rpoN downstream for rpoN deletion |
| rpoNR-R | gtgaacgccgccgcgtcg | Amplification of rpoN downstream for rpoN deletion |
| wzcL-F | cgcatcattgcataatctttcgc | Amplification of wzc upstream for wzc deletion |
| wzcL-R | ggtcgacggatccccggaatattgcggctgactatccgga | Amplification of wzc upstream for wzc deletion |
| aprR-F(wzc) | tccggatagtcagccgcaatattccggggatccgtcgacc | Amplification of apramycin cassette |
| aprR-R(wzc) | ctcaaagagcttatgcgcatatcctgtaggctggagctgcttc | Amplification of apramycin cassette |
| wzcR-F | gaagcagctccagcctacaggatatgcgcataagctctttgag | Amplification of wzc downstream for wzc deletion |
| wzcR-R | tgacattcttgacgagaagcctc | Amplification of wzc downstream for wzc deletion |
| wbaPL-F | cggtgtttgttgtgtaggctg | Amplification of wbaP upstream for wbaP deletion |
| wbaPL-R | ggtcgacggatccccggaatgaggcattccacgcaatgttg | Amplification of wbaP upstream for wbaP deletion |
| aprR-R(wbaP) | caacattgcgtggaatgcctcattccggggatccgtcgacc | Amplification of apramycin cassette |
| aprR-R(wbaP) | gcaattcatccaggcttgtccttgtaggctggagctgcttc | Amplification of apramycin cassette |
| wbaPR-F | gaagcagctccagcctacaaggacaagcctggatgaattgc | Amplification of wbaP downstream for wbaP deletion |
| wbaPR-R | gcttcgttacgagttccacca | Amplification of wbaP downstream for wbaP deletion |
| wza-conf-F | aacatgggagagaaggtggt | Confirmation of wza deletion |
| wza-conf-R | ttcgtaccagccgaatcaatc | Confirmation of wza deletion |
| wzc-conf-F | aaccagcgagatatcccaga | Confirmation of wzc deletion |
| wzc-conf-R | tcttgacgagaagcctcatg | Confirmation of wzc deletion |
| wbaP-conf-F | ggcgtggtgaagaaagctag | Confirmation of wbaP deletion |
| wbaP-conf-R | cggtagtacgccacaaatcc | Confirmation of wbaP deletion |
| rpoN-conf-F | gcagcagatcctcgaagatg | Confirmation of rpoN deletion |
| rpoN-conf-R | attgctccagcttgctgaac | Confirmation of rpoN deletion |
| pspA-rt-F | cgtctgatgatccaggagat | qRT-PCR primers for pspA |
| pspA-rt-R | tcggctttttcctgccattc | qRT-PCR primers for pspA |
| pspB-rt-F | gagcatgctttttctcgcca | qRT-PCR primers for pspB |
| pspB-rt-R | gccgttgctgctcattttgc | qRT-PCR primers for pspB |
| pspC-rt-F | aaactgtggcgtatcccgca | qRT-PCR primers for pspC |
| pspC-rt-R | agaggccgaaaatcatcgcc | qRT-PCR primers for pspC |
| pspD-rt-F | atagcaaatggcaacgcgcc | qRT-PCR primers for pspD |
| pspD-rt-R | gccagcagcattttcagcgg | qRT-PCR primers for pspD |
| pspG-rt-F | gtatcgctgctggggattat | qRT-PCR primers for pspG |
| pspG-rt-R | gccacggcagtaatttgatca | qRT-PCR primers for pspG |
| gapA-rt-F | gaaaggcgttctgggttac | qRT-PCR primers for reference gene gapA |
| gapA-rt-R | gatgtgggcaatcagatcc | qRT-PCR primers for reference gene gapA |
| wza-ntrd-1-F | gttatggatgcaataaatgc | Confirmation of the IS type in wza in KP10042 ntrd-1 |
| wza-ntrd-1-R | tgattttgtgttaaatcccc | Confirmation of the IS type in wza in KP10042 ntrd-1 |
| wzc-ntrd-2-F | caatctgcacctgaaatagc | Confirmation of the IS type in wzc in KP10042 ntrd-2 |
| wzc-ntrd-2-R | ctagcaacctagccaacc | Confirmation of the IS type in wzc in KP10042 ntrd-2 |
| wzc-ntrd-3-F | aaacttgactcttacaggag | Confirmation of the IS type in wzc in KP10042 ntrd-3 |
| wzc-ntrd-3-R | gctaaactctttgcatcttg | Confirmation of the IS type in wzc in KP10042 ntrd-3 |
| wza-ntrd-4-F | gtttacgtgacaggcgaagt | Confirmation of the IS type in wza in KP10042 ntrd-4 |
| wza-ntrd-4-R | acattacgccaatctgcgtc | Confirmation of the IS type in wza in KP10042 ntrd-4 |
| wzc-ntrds-1-F | gagttgggtataaatgtttatgc | Confirmation of the IS type in wzc in KP10042 ntrds-1 |
| wzc-ntrds-1-R | aagtttgaggctcggattg | Confirmation of the IS type in wzc in KP10042 ntrds-1 |
| wbaP-ntrds-3-F | atgtcattcatattcagtcatg | Confirmation of the IS type in wbaP in KP10042 ntrds-3 |
| wbaP-ntrds-3-R | atagacaatgagcctataatatc | Confirmation of the IS type in wbaP in KP10042 ntrds-3 |
| rpoN-F | ctgctgctgctctacgatgc | Confirmation of the IS type in rpoN in all KP10042 msdw and ntrds |
| rpoN-R | gttgcttgagcaaaccgatcttc | Confirmation of the IS type in rpoN in all KP10042 msdw and ntrds |
