# Supplementary material for: Genetic, virulence, and antimicrobial resistance characteristics associated with distinct morphotypes in ST11 carbapenem-resistant Klebsiella pneumoniae
Source: Virulence. 2024 May 12;15(1):2349768. doi: 10.1080/21505594.2024.2349768 (PMC11093053; doi:10.1080/21505594.2024.2349768)
Supplement: Supplemental Material [file KVIR_A_2349768_SM6163.zip › Table S2 competition.docx]

**Table S2. *In vitro* competition index (CI) of the different morphotypes**

| Morphology | Replicates | | | Average |
| --- | --- | --- | --- | --- |
|  | 1 | 2 | 3 |  |
| mcsw (CFU) | 11 | 21 | 15 | 16 |
| ntrd (CFU) | 65 | 91 | 72 | 76 |
| CI | 0.17 | 0.23 | 0.21 | 0.21 |
|  |  |  |  |  |
| mcsw (CFU) | 97 | 62 | 106 | 88 |
| msdw (CFU) | 0 | 0 | 0 | 0 |
| CI | - | - | - | - |
|  |  |  |  |  |
| mcsw (CFU) | 68 | 59 | 92 | 73 |
| ntrds (CFU) | 17 | 18 | 26 | 20 |
| CI | 4 | 3.28 | 3.54 | 3.65 |
|  |  |  |  |  |
