# Supplementary figures and images for: Genetic, virulence, and antimicrobial resistance characteristics associated with distinct morphotypes in ST11 carbapenem-resistant Klebsiella pneumoniae
Source: Virulence. 2024 May 12;15(1):2349768. doi: 10.1080/21505594.2024.2349768 (PMC11093053; doi:10.1080/21505594.2024.2349768)

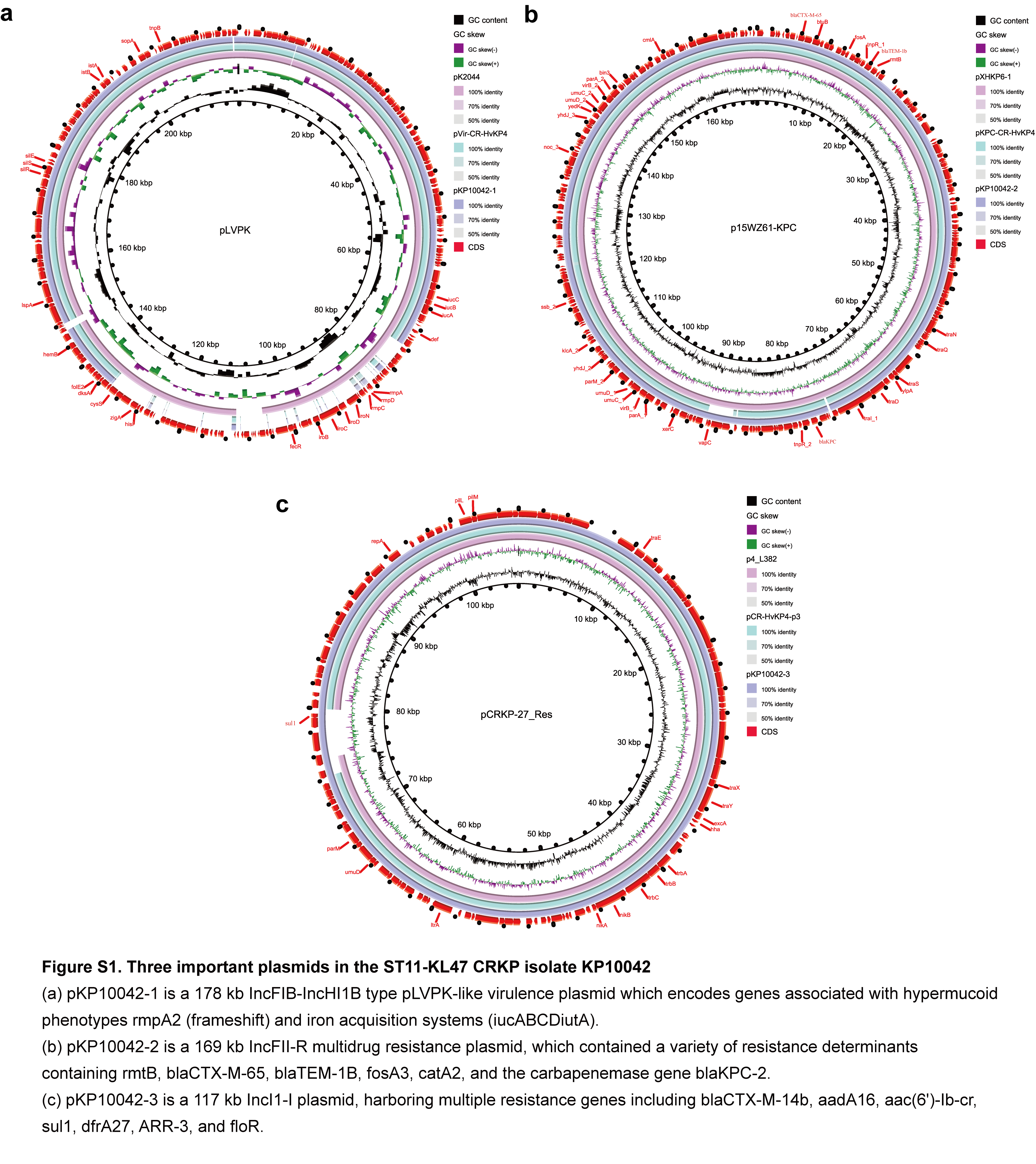

Supplement: Supplemental Material [file KVIR_A_2349768_SM6163.zip › FigS1.tif]

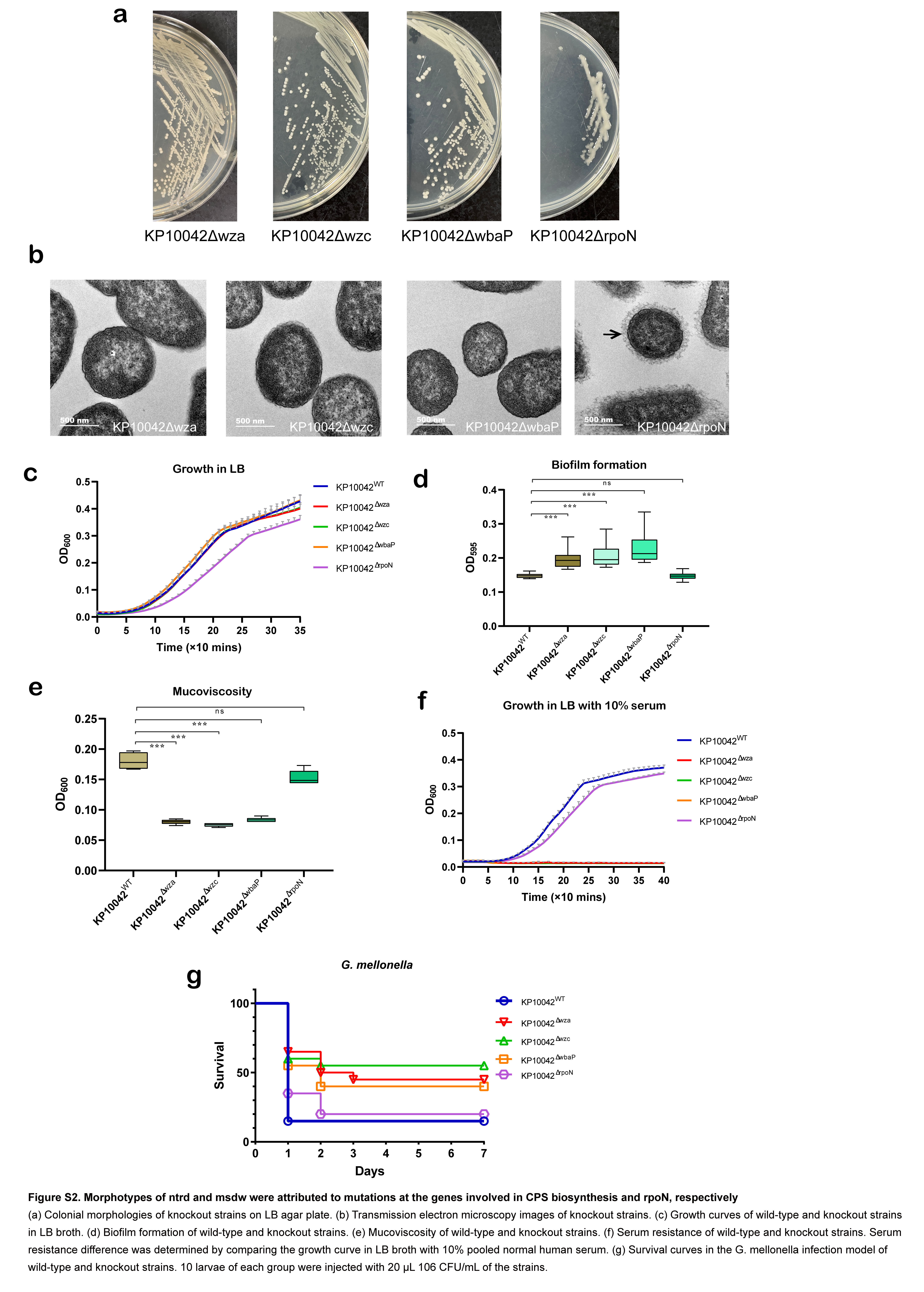

Supplement: Supplemental Material [file KVIR_A_2349768_SM6163.zip › FigS2.tif]
